# Survival status and its predictors among undernourished children on antiretroviral therapy in Bahir Dar city, Northwest Ethiopia, 2010 – 2020, a multicenter retrospective cohort study

**DOI:** 10.1186/s12887-024-04745-8

**Published:** 2024-04-30

**Authors:** Fikre Moga Lencha, Hailemariam Mekonnen Workie, Fikir Tadesse Mequanint, Zenebe Jebero Zaza

**Affiliations:** 1 School of Nursing, College of Medicine and Health Sciences, Arba Minch University, P.O. Box 21, Arba Minch, Ethiopia; 2 School of Health Sciences, Bahir Dar University, P.O. Box 79, Bahir Dar, Ethiopia

**Keywords:** Survival, Undernutrition, Children, HIV/AIDS, Predictors

## Abstract

**Background:**

In environments with limited resources, undernutrition is a serious public health risk. Its dual relationship to human immunodeficiency virus infection (HIV) leads to crises in a child's physical, emotional, social, and economic spheres of life. Nevertheless, little research has been done on the survival rate and risk factors that lead to poor survival outcomes in undernourished children receiving antiretroviral therapy. This study sought to evaluate survival status and its predictors among undernourished children on antiretroviral therapy (ART) in public health facilities, Bahir Dar city, September 1, 2010 – December 31, 2020.

**Methods:**

An institution-based retrospective cohort study design was used among 414 study participants from September 1, 2010 – December 31, 2020. A simple random sampling method was applied to select study participants. All collected data were entered into epi data version 4.6 and exported to STATA version 14.0 for analysis. Each independent predictor variable with a *p*-value < 0.05 in the multivariable Cox proportional hazard regression was considered statistically significant.

**Results:**

The overall incidence of mortality was 11.6 deaths per 1000 child year observation (95%CI: 7.7- 17.5). Baseline weight for age < -3 Z score (adjusted hazard ratio (AHR) = 4.9, 95% CI: 1.30–18.98), height for age < -3 Z score (AHR = 4.34, 95%CI 1.13–16.6), cotrimoxazole prophylaxis given (AHR = 0.27, 95%CI 0.08–0.87), hemoglobin level < 10 g/dl (AHR = 3.7, 95%CI 1.1–12.7), CD4 cells < threshold (AHR = 4.86, 95%CI 1.9–12.7), and WHO clinical disease stage III and IV (AHR = 8.1, 95%CI 1.97–33) were found independent predictors of mortality.

**Conclusion and recommendation:**

The incidence of mortality was determined in the study to be 11.6 per 1000 child years. Mortality was predicted by severe stunting, severe underweight, a low hemoglobin level, a low CD4 count, and WHO clinical stages III and IV. But the risk of death is reduced by starting cotrimoxazole preventative therapy early. The risk factors that result in a low survival status should be the primary focus of all concerned bodies, and early cotrimoxazole preventive treatment initiation is strongly recommended.

## Introduction

Undernutrition is defined as an insufficient intake of energy and nutrients to meet an individual’s needs to maintain optimum health [1]. It is the consequence of an insufficient intake of macronutrients and/or micronutrients, poor absorption or rapid loss of nutrients due to increased energy expenditure or illness and manifests in four broad formation: stunting, underweight, wasting, and micronutrient deficiencies [2]. According to World Health Organization (WHO), low weight for age is referred to as underweight. Underweight children may be wasted, stunted, or both [3]. Being low weight relative to height is called wasting. It might also last for a very long period. Usually, it signifies recent and significant weight reduction [3, 4]. The definition of stunting is low height for age. It is brought on by persistent or recurring undernutrition, which is typically linked to poverty, a high rate of disease, and/or improper early nutrition and care [4].

The burden of undernutrition among children on antiretroviral-therapy (ART) remains public health concern and the pathophysiologic effect of undernutrition results in poor absorption of ART from the gut and leads to inadequate concentration of ART which may lead to unpleasant health outcome including death [5, 6].

Besides, its effect among children with HIV infection is devastating and has increased the risk of mortality even after nutritional and medical interventions. In addition to this, it has adverse consequences in later life such as; low intellectual ability, low work productivity, poor school achievements, and being in poverty [7, 8].

Despite the fact that ART has saved millions of children from death, studies have revealed a short survival time among undernourished children compared to well-nourished children in resource-limited settings [9]. Also, undernutrition is not confined to resource-limited settings; it has been noted in chronic infections like HIV anywhere in the world [10].

In Sub-Saharan Africa, including Ethiopia, the magnitude of undernutrition has increased since 2015 from 17 to 22%, and HIV/AIDS-related burdens remain high [11, 12]. In Nigeria, the prevalence of being underweight, stunting, and wasting among HIV-positive children under the age of five was 77%, 65%, and 63%, respectively [13]. In a Cameroonian hospital, undernutrition was 68.7% among children with HIV [14]. In Northwest Ethiopia, 64% of children with HIV infection were undernourished [15].

Due to an imbalance between the body’s calorie requirement and daily calorie intake among children with HIV infection, children are likely to rapid disease progression, increased morbidity, and reduced survival [16, 17]. Different studies have shown predictors of mortality among HIV positive children. Low CD4 count/below thresh hold at the time of ART initiation, advanced WHO stage of disease, compromised nutritional status, poor adherence to ART are some of the independent predictors of mortality [5, 6, 18, 19].

In order to end undernutrition by 2030, the Ethiopian government runs initiatives like the Sustainable Undernutrition Reduction in Ethiopia, which took the place of the previous community-based nutrition program and works in partnership with different concerned organizations like UNICEF, Nutrition International, and a significant number of others [20–22].

The efforts made by Ethiopian government has showed consistent economic growth, significant pro-poor spending, and appreciable agriculture and health extension networks that collectively have resulted in a remarkable reduction in undernutrition [23]. Nevertheless, the alarmingly high (28%) magnitude of child mortality linked to undernutrition persists [21].

Furthermore, in Ethiopia, the number of children to be treated for severe undernutrition in 2020 alone has increased by 24% due to the effects of desert locusts, the COVID-19 pandemic, ongoing and severe droughts, climatic change, and internal displacements [24]. A rise of this magnitude will surely result in child mortality, especially in children with chronic diseases, HIV/AIDS, and recurrent infections who are likely to die from undernutrition [22, 24]. Therefore, this study aimed to assess survival status and its predictors among undernourished children on ART in public health facilities, Bahir Dar city, Northwest Ethiopia, September1, 2010 – December 31, 2020.

## Methods and materials

### Study area and study period

Bahir Dar city is the administrative capital of Amhara regional state and located 565 km Northwest of Addis Ababa, Ethiopia’s capital city. The city has two referral hospitals, one primary hospital, and 10 health centers. From those public health facilities, one referral hospital, one primary hospital, and 8 health centers have been giving ART services. According to November 2019s Amhara regional health bureau report the people receiving ART are about 142,593 of these 5637 are children. The total number of children receiving ART from 2010 to 2020 accounts for 1621. Of these children, about 634 were undernourished at the initiation of ART. The chart review was held in Bahir Dar city from March 10 to April 10, 2021.

### Study design

A Facility based retrospective cohort study design was used.

### Source population

All undernourished children less than 15 years old who initiated ART in public health facilities, Bahir Dar city.

### Study population

All undernourished children under 15 years old who received ART in Bahir Dar city public health facilities between September 1, 2010, and December 31, 2020. We only considered under fifteen children because children aged 15 and above were considered adults according to the national consolidated ART care, prevention, and treatment guidelines.

### Inclusion and exclusion criteria

#### Inclusion criteria

All undernourished children who were on ART from September1, 2010 to December31, 2020.

#### Exclusion criteria

Patients with incomplete baseline information’s (like; the child’s age, baseline nutritional status, initiation date of ART and exit date from the cohort).

#### Study variables

##### Outcome variable

Death.

##### Independent variables

Socio demographic Factors: Age, Sex, Residence, Caregiver Relationship, Caregiver HIV Status, Parental Live Status, Caregiver Marital Status, and Caregiver Occupational Status. Baseline clinical and lab-related factors: WHO clinical stage, hemoglobin count, CD4 count, opportunistic infection, child’s function status, developmental status, nutritional status, IHN prophylaxis, and CPT prophylaxis. ART-related factors: Adherence to ART drugs, time of initiation, and regimen-related.

### Sample size determination

By utilizing the double population proportion formula, along with the assumptions of a 95% confidence interval, 80% power, and a 1:1 ratio of exposed to unexposed individuals, the sample size was determined using Epi Info software. From the explanatory variables, height for age (normal/unexposed versus severely stunted/exposed), with 23.4% outcome among exposed and 4.5% outcome among unexposed, gave a sample size of 180. Again, weight for height (normal/unexposed versus severely wasted/exposed), with a 27.8% outcome among exposed and a 6.27% outcome among unexposed, gave a sample size of 218. The CD4 count outcome, with 12% of individuals falling below the threshold (exposed) and 4.3% above the threshold (unexposed), gave a sample size of 396. Further, many other variables other than the aforementioned were considered, but they gave a sample of small size. Based on this, the maximum required sample size was calculated to be 396 [25], and an additional 10% was added to account for incomplete charts, resulting in a final sample size of 436.

### Sampling procedure and sampling technique

**Fig. 1:**
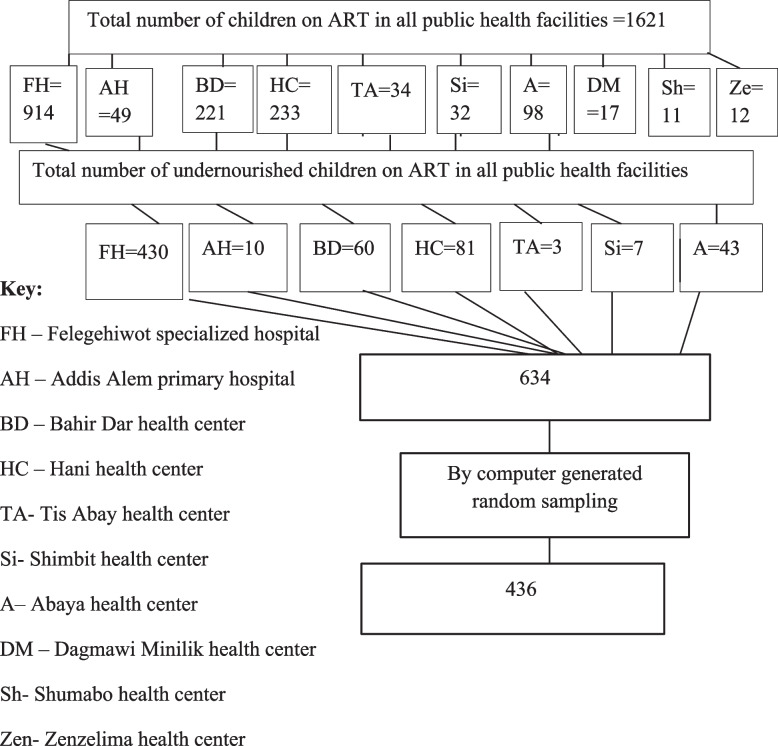
Diagrammatic representation of sampling procedure to assess survival status and its predictors among undernourished children on ART in public health facilities, Bahir Dar city from September 1, 2010 – December 31, 2020

This procedure took into consideration the uniformity of ART-standard services offered by all public health facilities. All public health facilities provided the medical registration numbers (MRN) of the undernourished children, which were then combined and input into Microsoft Excel. After importing these data into the Statistical Package for the Social Sciences (SPSS) software, a random sample of 436 undernourished children out of the 634 total undernourished children across eleven public health facilities was created. Finally, the sample unit was reached by using a MRN (Fig. 1).

### Operational definitions

*Survival status*- Defined as the length of time from ART initiation to death or censure.

*Undernutrition*- Child having height for age < -2 Z score, and/or weight for age < -2 Z score, and/or Weight for height/length < -2 Z score according to (2006) WHO standard curve [4].

*ART Adherence*- For children is classified based on the percentage of ART dosage calculated from the total monthly doses of ART drugs: good > 95%, fair = 85 − 94%, and poor < 85% [26].

*Censored*- Children alive beyond the study period, lost to follow up and transfer out to other health facility.

*Developmental miles*- Were described as appropriate, delayed, and regressive for children under the age of five [27].

*Developmentally appropriate*- Refers to the child having achieved all domains of development milestones for his or her age.

*Developmentally delayed*- Means when a child is slowed to reach one or more developmental milestones compared to his/her peers.

*Developmentally regressive*- This is the loss of acquired milestones in a normally developing child who has reached all developmental milestones.

*Functional status*- Was expressed as working (go to school, do normal activity, etc.), ambulatory (able to perform routine daily activities), and bedridden (not able to perform routine daily activities) for children aged five and above [27].

### Data collection tools and procedures

Data were extracted through chart review using structured checklists that were adapted from ART intake and follow-up forms after reviewing different kinds of literature [6, 18, 25, 28–31]. The data extraction checklist comprises socio-demographic characteristics, which contained nine questions; baseline clinical, laboratory, and ART information, which in general had twenty-five questions; and the outcome variable (dead, dropout, transfer out, and alive on ART) section, which had six questions. The starting point for retrospective follow-up for each subject was from the date of ART initiation to the date of death if died, from the date of ART initiation to the date of the final visit if lost to follow-up, and from the date of ART initiation to the Alive on ART if alive at the end of the study. The WHO Anthro and AnthroPlus software for health data was used to gather information about the nutritional status and evaluate the children's growth and development for their age.

### Data quality assurance

Prior to the actual data collection, a pretest involving 5% of the sample population was carried out to assess the checklists' consistency. Then variables in the tools that could have been incomplete were eliminated, considering the pretest results. Two days of training were given to both the supervisor and the data collectors. One BSc. nurse oversaw the data gathering process, while three diploma nurses collected the data. Before data analysis, the proper data entry methods were used with epi-data version 4.6. The frequency and completeness of the data were checked before exporting to Stata for further analysis.

### Data processing and analysis

The entered data into epi-data software version 4.6 was exported to Stata version 14.0 for analysis. The socio-demographic and clinical features were described for the entire cohort by using frequencies for categorical variables. The mean with standard deviation were used to describe the characteristics of continuous variables. The outcome variable was dichotomized into censored and death. Before running the Cox regression model, multi-collinearity and interaction terms were checked by using the variance inflation factor (VIF), which gave an overall mean value of 1.74 and a mean value less than 10 for each independent variable. Cox proportional hazard model assumptions were checked by using a global test and graphically by using the Kaplan–Meier versus predicted survival plot of the categorical independent variables. The global test *P*-value greater than 0.05 shows the Cox proportional hazard model assumption was satisfied. Each independent predictor variable with a *p*-value < 0.2 in the bivariable analysis was included in the multivariable Cox proportional hazard regression model. In the multivariable Cox regression model, variables with a *P*-value < 0.05 were considered statistically significant. Differences in overall survival probability curves were displayed by using Kaplan–Meier plots, and a log-rank test was used to compare the survival time between different categories of explanatory variables. The hazard ratio and *p*-value were used to show the level of association between explanatory variables and an outcome variable.

## Results

### Baseline socio demographic characteristics of undernourished children on ART

Of the total 436 charts reviewed, 414 charts of undernourished children on ART were included in the analysis, which gave a completeness rate of 94.95%. Of those children, 220 (53.14%) were males, and 342 (82.61%) resided in urban areas. The mean age of children was 7.63 ± 0.2 years, and above two-thirds (69.81%) of children were following ART care services in hospitals. Regarding the incidence of death, the majority (52.2%) of deaths occurred among female children, and 35% of deaths occurred among adolescents aged 10 and less than 15 years. Around ¾ of deaths occurred among urban residents, and 87% of deaths occurred among children following ART services in hospitals (Table 1).

**Table 1:** Baseline socio demographic characteristics of undernourished children on ART in public health facilities, Bahir Dar city, Northwest Ethiopia, September 1, 2010—December 31, 2020

Variable	Frequency (%)	Death (%)	Survived (%)	*P*-value
Sex of child				
Male	220(53.14)	11(47.80)	209(53.50)	0.56
Female	194(46.86)	12(52.20)	182(46.50)	
Age of child				
< = 1 year	29(7.00)	3(13.00)	26(6.70)	0.24
2–5 years	93(22.46)	7(30.00)	86(22.00)	
5–10 years	149(36.00)	5(22.00)	144(36.80)	
10–15 years	143(34.54)	8(35.00)	135(34.50)	
Residence of child				
Urban	342(82.61)	17(74.00)	325(83.00)	0.26
Rural	72(17.39)	6(26.00)	66(17.00)	
Follow up place				
Health center	125(30.19)	3(13.00)	122(31.00)	0.08
Hospital	289(69.81)	20(87.00)	269(69.00)	

### Baseline socio demographic characteristics of children’s primary care givers information

Of the total, 370 (89.37%) of children’s primary caregivers were parents and about (66.91%) of caregivers were married. Regarding to parental status of child, almost sixty six percent (65.7%) of parents were alive and around twenty nine percent 122 (29.47%) of children had at least one parent died. Concerning to occupational status of care givers, more than one fourth (25.79%) of care givers were house wife and around twenty two percent (22.14%) were merchants. The majority (56.6%) of deaths recorded were among children who had both alive parents, and approximately 83% of dead children live with one or both of their parents. The majority of primary caregivers of deceased children were married, and 74% of deaths happened among children living with HIV-positive caregivers. The majority (26.1%) of deaths were recorded among the children of merchants (Table 2).

**Table 2:** Baseline socio demographic characteristics of children’s primary care givers in public health facilities, Bahir Dar city, Northwest Ethiopia, September1, 2020 – December 31, 2020

Variable	Frequency (%)	Death (%)	Survived (%)	*P*-value
Parental status				
Both alive	272(65.70)	13(56.60)	259(66.30)	0.67
Father died	81(19.57)	5(21.70)	76(19.40)	
Mother died	41(9.90)	3(13.00)	38(9.70)	
Both died	20(4.83)	2(8.70)	18(4.60)	
Primary care giver relation				
Parents	370(89.37)	19(82.60)	351(89.70)	0.53
Siblings	14(3.38)	2(9.00)	12(3.00)	
Uncles/aunts	3(0.72)		3(0.80)	
Grandparents	10(2.42)	1(4.30)	9(2.30)	
Others	17(4.11)	1(4.30)	16(4.10)	
Marital status of care giver(*N* = 405)				
Single	22(5.43)	2(8.70)	20(5.20)	0.55
Married	271(66.91)	12(52.20)	259(67.80)	
Divorced	28(6.91)	2(8.70)	26(6.80)	
Widowed	84(70.74)	7(30.40)	77(20.20)	
Care giver’s HIV status				
Positive	337(81.40)	17(74.00)	320(81.80)	0.11
Negative	22(5.31)	3(13.00)	19(4.90)	
Unknown	55(13.29)	3(13.00)	52(13.30)	
Occupational status (*N* = 411)				
Gov’t worker	85(20.68)	3(13.00)	82(21.10)	0.82
House wife	106(25.79)	4(17.40)	102(26.30)	
Merchant	91(22.14)	6(26.10)	85(21.90)	
Farmer	29(7.06)	2(8.70)	27(6.96)	
Daily laborer	37(9.00)	3(13.00)	34(8.76)	
Drivers	50(12.17)	4(17.40)	46(11.86)	
Others	13(3.40)	1(4.30)	12(3.10)	

### Baseline clinical and laboratory profiles

**Fig. 2:**
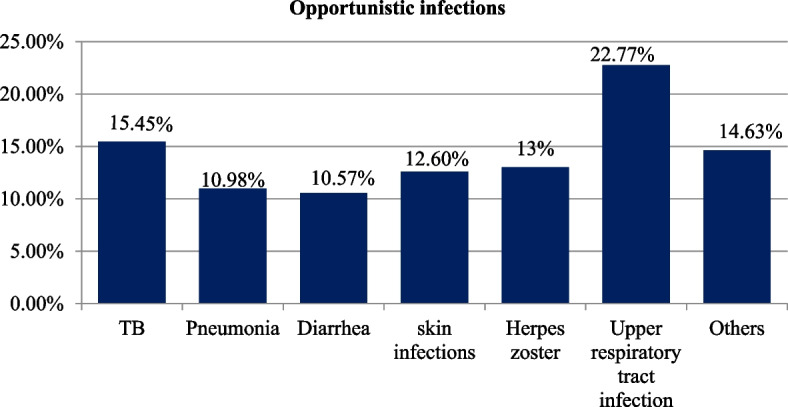
Baseline opportunistic infections among undernourished children on ART in public health facilities, Bahir Dar city, Northwest Ethiopia, September1, 2010 – December 31, 2020

From the total, approximately sixty percent 246 (59.42%) of children had at least one opportunistic infection(OI) at time of ART initiation and of these opportunistic infections the most common one was upper respiratory tract infections (URTI’s) which accounts around twenty three percent 56 (22.77%) (Fig. 2).

The majority (69.08%) of children were in WHO clinical stages I and II, and about 82.78% of participant children had CD4 count greater than or equal to threshold. Concerning nutritional status; about 145 (35.02%) of children had moderate stunting, about 108 (26.08%) of children had moderate underweight, and above half (55.56%) of children had no wasting. The majority (95.54%) of children had baseline hemoglobin levels greater than or equal to 10 g/dl, and the majority (87.65%) of children had a viral load fewer than 1000 copies. About 338 (81.64%) of children had baseline cotrimoxazole preventive treatment history, and more than sixty one percent (61.11%) of children had no history of baseline isoniazid preventive treatment. Regarding developmental milestones, about 104 (85.25%) of under-five children had appropriate developmental miles for their age, and regarding to the functional status of five and above five years old children, the majority (92.81%) of the children were working. In regard to death, approximately 78% of deaths among children had one or more opportunistic infections, and 69.6% of deaths recorded among children were in WHO stages III and IV. The majority (80%) of deaths occurred among under-five children who had appropriate development for their age, and approximately 85% of deaths happened among children older than five years who were working. The majority (52%) of deaths were recorded among children who had a CD4 count lower than the threshold, and 30.4% of the deaths occurred among children who had a hemoglobin level less than 10 g/dl. About 65.2%, 78.3%, and 43.5% of deaths occurred among children who were severely underweight, severely stunted, and severely wasted, respectively. Regarding IHN and cotrimoxazole preventive therapy, 82.6% of deaths happened among children who didn’t take IHN prophylaxis, and 78.3% of deaths occurred among children who took cotrimoxazole prophylaxis (Table 3).

**Table 3:** Baseline clinical, and laboratory profiles of undernourished children on ART in public health facilities, Bahir Dar city, Northwest Ethiopia, September, 2010 – December, 2020

Variable	Frequency (%)	Death (%)	Survived (%)	*P*-value
Opportunistic infections (*n* = 414)				
Yes	246(59.42)	18(78.30)	228(58.30)	0.08
WHO stage				
S I &SII	286(69.08)	7(30.40)	279(71.35)	0.0001
Stage III&IV	128(30.92)	16(69.60)	112(28.650)	
Developmental history(< 5 years)(*n* = 122)				
Appropriate	104(85.25)	8(80.00)	96(85.70)	0.09
Delayed	17(13.93)	2(20.00)	15(13.40)	
Regressive	1(0.82)	-	1(0.89)	
Functional status (> 5 years)(*n* = 292)				
Working	271(92.81)	11(84.60)	257(93.10)	0.05
Ambulatory	19(6.51)	1(7.70)	18(6.50)	
Bedridden	2(0.68)	1(7.70)	1(0.36)	
CD4 count (*N* = 396)				
< Threshold	69(17.40)	12(52.20)	56(15.00)	0.0001
≥ Threshold	327(82.60)	11(47.80)	317(85.00)	
Hemoglobin level (*N* = 404)				
< 10 g/dl	20(4.95)	7(30.40)	13(3.40)	0.001
≥ 10 g/dl	384(95.05)	16(69.60)	368(96.60)	
Viral load(*N* = 405)				
< = 1000	355(87.65)	18(78.30)	337(88.20)	0.28
> 1000	50(12.35)	5(21.70)	45(11.80)	
Weight/Age				
> = -2 Z score	214(51.70)	5(21.70)	209(53.50)	0.0001
< -2 Z score	108(26.08)	3(13.10)	105(26.90)	
<—3 Z score	92(22.22)	15(65.20)	77(19.70)	
Height/Age				
> = -2 Z score	142(34.30)	4(17.40)	138(35.30)	0.0001
< -2 Z score	145(35.02)	1(4.30)	144(36.80)	
<—3 Z score	127(30.67)	18(78.30)	109(27.90)	
Weight/H/L				
> = -2 Z score	230(55.56)	10(43.50)	220(56.30)	0.009
< -2 Z score	103(24.88)	3(13.00%)	100(25.60)	
<—3 Z score	81(19.56)	10(43.50)	71(18.10)	
IHN prophylaxis				
Yes	161(38 .89)	4(17.40)	157(40.15)	0.44
Cotrimoxazole Prophylaxis				
Yes	338(81.64)	18(78.30)	320(81.80)	0.03

### Antiretroviral therapy related factors

Regarding ART eligibility criteria, above half (53.62%) of children were eligible by both WHO clinical stage and immunological. Concerning the ART initiation time, about 211 (50.97%) of children initiated ART immediately within seven days of eligibility. The majority (65.22%) of participants had initial ART change history and a total of 245 (90.74%) of changes were within the first line. A frequent reason (41.85%) for change was the availability of new drugs, and about 77 (18.6%) of children had drug side effect reports. About 93% of children had good adherence to ART at first three months. In relation to death, approximately 74% of deaths were recorded among children who had no reports of ART drug side effects. The majority (78.3%) of deaths occurred among children who were eligible for ART by both CD4 count and WHO clinical stage criteria and 52.2% of deaths occurred among children who started ART on the 7th day or longer after ART eligibility. Approximately 60% of deaths were recorded among children who had no ART regimen change, and 87% of deaths occurred among children who had good ART adherence (Table 4).

**Table 4:** ART related factors among undernourished children on ART in public health facilities, Bahir Dar city, Northwest Ethiopia, September1, 2010 – December 31, 2020

Variables	Frequency (%)	Death (%)	Survived (%)	*P*-value
Drug side effect(*n* = 414)				
Yes	77(18.60)	6(26.10)	71(18.20)	0.34
ART eligibility criteria				
Immunologic	96(23.19)	4(17.40)	92(23.50)	0.10
WHO stage	38(9.18)	1(4.30)	37(9.50)	
Both	222(53.62)	18(78.30)	204(52.20)	
Without criteria	58(14.01)	-	58(14.83)	
Date b/n ART eligibility—initiation				
< 7 days	211(50.97)	11(47.80)	200(51.20)	0.90
≥ 7 days	203(49.03)	12(52.20)	191(48.80)	
Regimen change				
Yes	270(65.22)	9(39.10)	261(66.75)	0.006
Change was ( *N* = 270)				
Within first line	245(90.74)	7(77.77)	238(91.20)	0.12
To second line	25(9.26)	2(22.23)	23(8.80)	
Reason for change				
Drug side effect	76(28.15)	4(44.44)	72(27.60)	0.81
OI	4(1.48)	1(11.11)	3(1.15)	
Treatment failure	20(7.41)	2(22.22)	18(6.90)	
Drug stock out	34(12.59)	-	34(13.00)	
New drug	113(41.85)	2(22.22)	111(42.50)	
Others	23(8.52)	-	23(8.80)	
Adherence				
Good	385(93.00)	20(87.00)	365(93.40)	0.0001
Fair	1(0.24)	1(4.3%)	-	
Poor	28(6.76)	2(8.70)	26(6.60)	

**Fig. 3:**
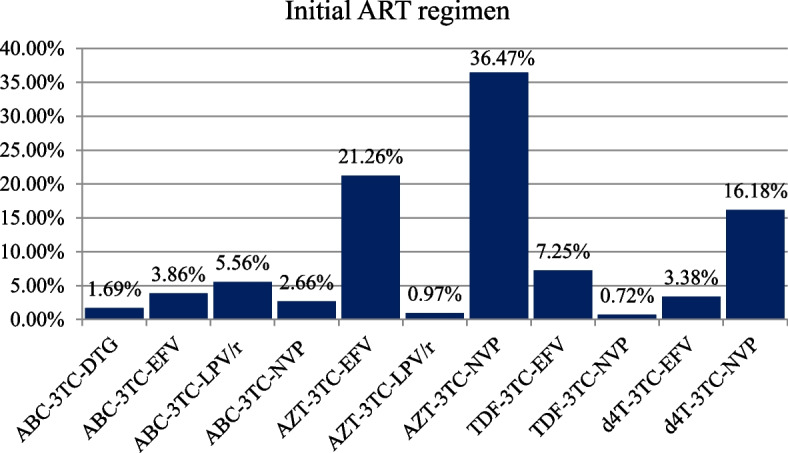
Initial ART regimen among undernourished children on ART in public health facilities, Bahir Dar city, Northwest Ethiopia, September 1, 2010 – December 31, 2020

Concerning the initial ART regimen, the majority (36.47%), 21.26%, and 16.18% of children initiated AZT-3TC-NVP, AZT-3TC-EFV, and d4T-3TC-NVP, respectively, and about 1.69, 0.97, and 0.72% of participants initiated ABC-3TC-DTG, AZT-3TC-LPV/r, and TDF-3TC-NVP, respectively (Fig. 3).

### Survival status of undernourished children on ART

**Fig. 4:**
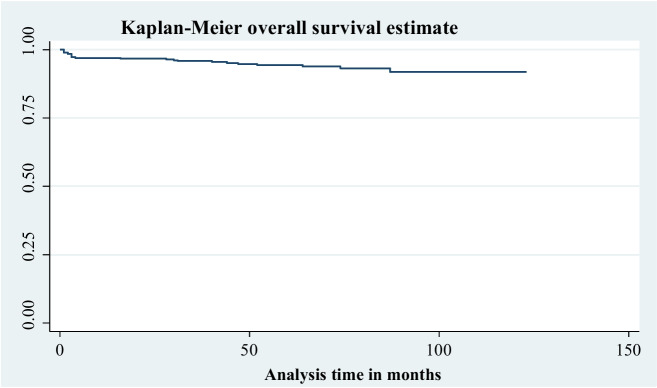
Kaplan Meier Estimate of an overall survival functions among undernourished children on ART in public health facilities, Bahir Dar city, Northwest Ethiopia, September 1, 2010 – December 31, 2020

This study followed for a minimum of one month and a maximum of one hundred twenty-three months. Within a median follow-up of 61 months, a total of 319 (77.05%) children were alive beyond the study period, 23 (5.56%) died (95% CI: 3.7–8.2), 6 (1.45%) dropped out, and 66 (15.94%) transferred out. Regarding time to death, 50%, 54.55%, and 68.18% of death occurred at twelve, twenty-four, and thirty-six months respectively. The mean survival time for the entire cohort was 116.1 months (95% CI: 113.35—118.85) with a standard deviation of 1.40 months. An estimated cumulative survival probability at 3, 6, 12, 24, 36, and 123 months were 0.985, 0.9704, 0.9704, 0.9677, 0.9587, and 0.9189 respectively. The overall incidence of death was 11.6 per 1000 child year observation (95% CI: 7.7—17.5). This entire cohort followed for a total of 23,760 months. An overall Kaplan Meier survival estimate showed that the incidence of death was highest at an early age (first 36 months) on ART compared to later ages on ART (Fig. 4).

### Comparison of survival curves over categorical variables

**Fig. 5:**
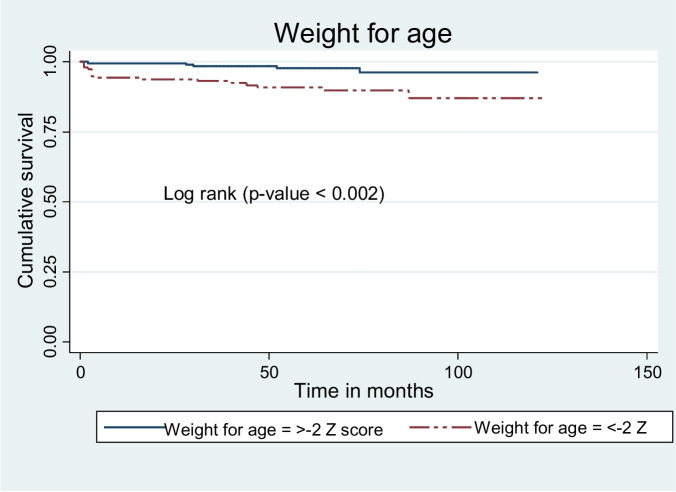
Kaplan Meier survival curves to compare undernourished children on ART with categories of weight for age in public health facilities, Bahir Dar city, Northwest Ethiopia, September 1, 2010 – December 31, 2020

As a log-rank test results showed significant differences exist among different explanatory variables. Among the variables, baseline nutritional status, baseline CD4 count (less than threshold versus greater than or equal to threshold), baseline hemoglobin level greater than or equal to 10 g/dl versus less than 10 g/dl, WHO clinical stage I and II versus stage III and IV, and initial regimen changed versus not changed. The mean survival time for participants with baseline underweight was 111.6 ± 2.6 months while the mean survival time for not underweight participants was 118.3 ± 1.3 months (Fig. 5).

**Fig. 6:**
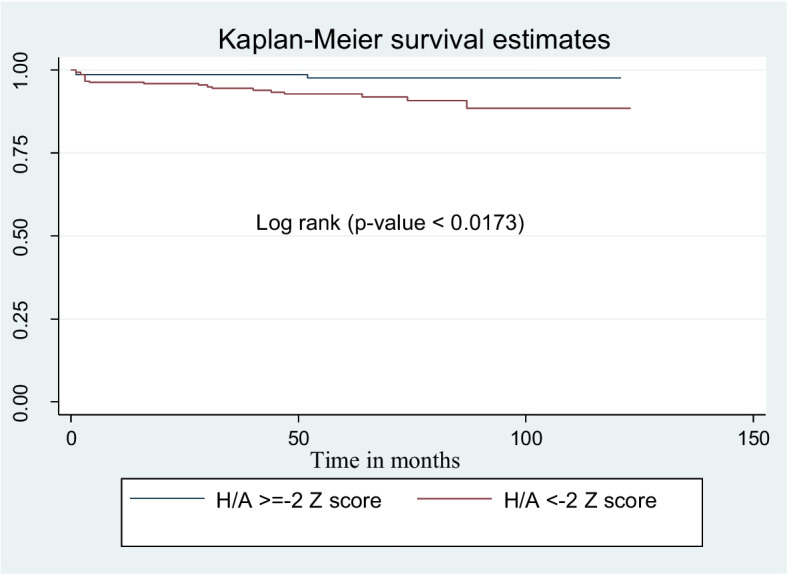
Kaplan Meier survival curves to compare undernourished children on ART with categories of height for age in public health facilities, Bahir Dar city, Northwest Ethiopia, September 1, 2010 – December 31, 2020

Children presented with baseline height for age (H/A) < -2 Z score had shorter survival mean time compared to participants presented with ≥ -2 Z score. The mean survival time was 113.5 ± 2 months for participants H/A < -2 Z score and 118.7 ± 1.3 months for participants H/A ≥ -2 Z score (Fig. 6).

**Fig. 7:**
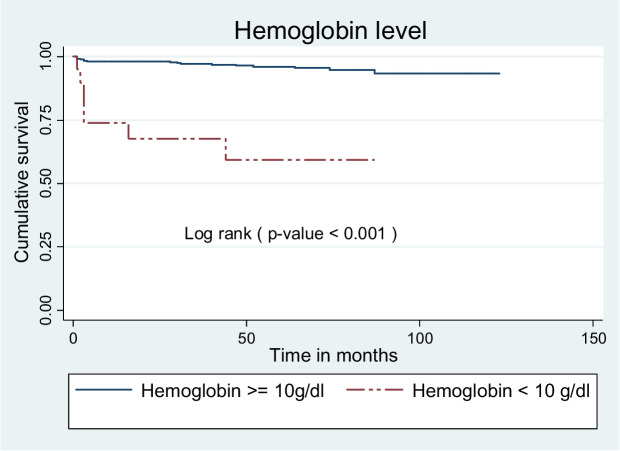
Kaplan Meier survival curves to compare undernourished children on ART with categories of baseline hemoglobin level in public health facilities, Bahir Dar city, Northwest Ethiopia, September 1, 2010 – December 31, 2020

Children presented with baseline hemoglobin level < 10 g/dl had shorter survival mean time compared to participants presented with ≥ 10 g/dl. The mean survival time was 113.8 ± 2 months for participants < 10 g/dl and 117.9 ± 1.5 months for participants ≥ 10 g/dl (Fig. 7).

**Fig. 8:**
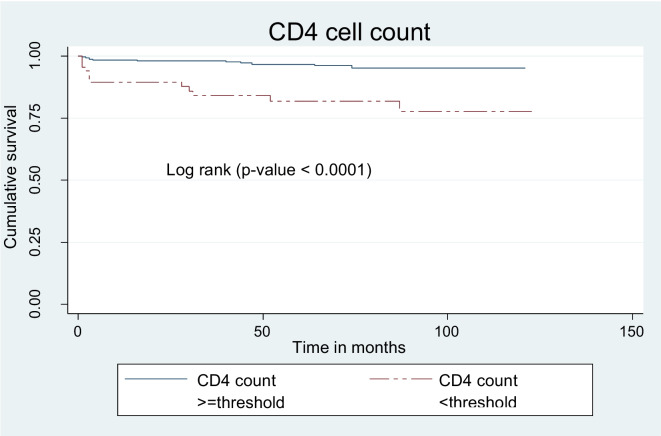
Kaplan Meier survival curves to compare undernourished children on ART with categories of baseline CD4 count in public health facilities, Bahir Dar city, Northwest Ethiopia, September 1, 2010 – December 31, 2020

The mean survival time for children presented with CD4 count below threshold was lower than for their counterpart children ≥ threshold (102 ± 5 months and 117 ± 1 months, respectively) (Fig. 8).

**Fig. 9:**
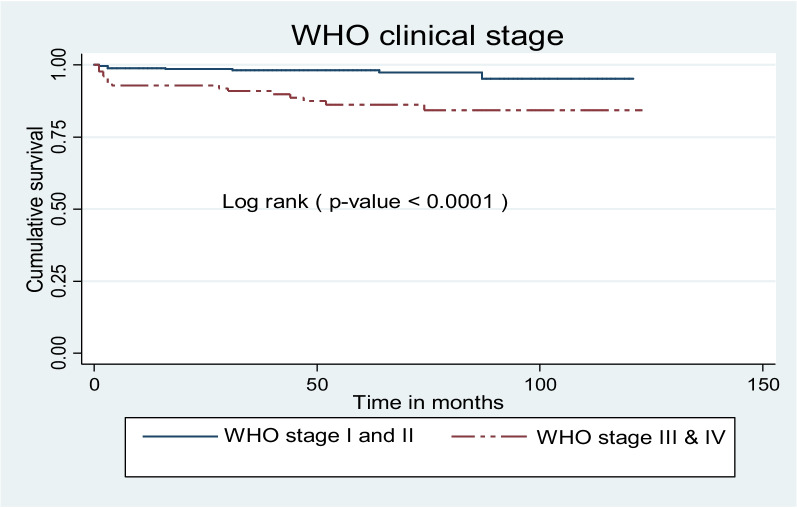
Kaplan Meier survival curves to compare undernourished children on ART with categories of baseline WHO clinical stage in public health facilities, Bahir Dar city, Northwest Ethiopia, September1, 2010 – December 31, 2020

An estimated mean survival time for children with advanced WHO clinical stage was 108 ± 3.5 months while 117.8 ± 1.2 months for those with mild WHO clinical stage presentation (Fig. 9).

### Predictors of mortality

Temporary relation between explanatory variables and death was analyzed using Cox proportional hazard regression model. In multivariable analysis, about six variables had a statistically significant relation to the risk of death among children. These are baseline weight for age (W/A) ≥ -2 Z score versus weight for Age (W/A) < 2 Z score, baseline height for age (H/A) ≥ -2 Z score Versus H/A <—2 Z score, baseline cotrimoxazole prophylaxis given versus not given, baseline hemoglobin level ≥ 10 g/dl versus hemoglobin level less than 10 g/dl, CD4 count ≥ threshold versus CD4 count less than threshold, WHO clinical stage I&II versus stage III & IV and regimen changed versus regimen not changed. As multivariable analysis result has shown, children with baseline weight for age (W/A) < -3 Z score were almost 4.9 times more likely to survive shorter duration as compared to children with baseline weight for age (W/A ≥ -2 Z score (AHR: 4.9, 95% CI: 1.3, 18.98) (*p*-value = 0.02). Children exposed to baseline stunting (H/A) < -3 standard Z score were 4.34 times at more risk to death as compared to children who didn’t expose to baseline stunting (H/A) ≥ -2 standard Z score (AHR = 4.34, 95%CI 1.13, 16.6) (*p*-value = 0.03). Regarding to cotrimoxazole prophylaxis, the estimated hazard of death among children who did take baseline cotrimoxazole prophylaxis properly was reduced by 73% as compared to their counterpart children who didn’t take baseline cotrimoxazole prophylaxis (AHR = 0.27, 95%CI 0.09, 0.87) (*p*-value = 0.03). Children presented with baseline hemoglobin level < 10 g/dl were almost 3.7 times more likely to survive a shorter duration than those children with baseline hemoglobin level ≥ 10 g/dl (AHR = 3.7, 95%CI 1.1, 12.7) (*p*-value = 0.037). In multivariable analysis, an estimated hazard of death was 4.86 times high among children with baseline CD4 < threshold as compared to those children with CD4 count ≥ threshold (AHR = 4.86, 95%CI 1.9, 12.7) (*p*-value = 0.001). Moreover, children with baseline WHO clinical disease stage (III and IV) were almost 8.1 times more risk to death than those children presented with WHO clinical stage I and II (AHR = 8.1, 95%CI 1.97, 33) (*p*-value = 0.004). Children who had a history of regimen change have a low risk of mortality compared to children with no regimen change history (AHR = 0.35, 95%CI 0.12, 1.00) (*p*-value = 0.05) (Table 5).

**Table 5:** Bivariate and Multivariable cox proportional hazard regression among undernourished children on ART in public health facilities, Bahir Dar city, Northwest Ethiopia, September 1, 2010 – December 31, 2020

Variable	Category	Survival status		CHR (95%CI)	AHR (95% CI)
		Death	Censored		
Opportunistic	Yes	18(7.32%)	228(92.68%)	2.3(0.86—6.26)	0.24(0.046–1.27)
Infection	No	5(3.07%)	163(96.93%)	1	1
Weight for age	≥ -2 Z score	5(2.33%)	209(97.67%)	1	1
	< -2- ≥ -3 Z score	3(2.78%)	105(97.22%)	1.2(0.3—5)	0.87(0.18—4.2)
	< -3 Z score	15(16.30)	77(83.7%)	7.76(2.8, 21.3)	4.9(1.3 -18.98)*
Height for age	≥ -2 Z score	4(2.82%)	138 (97.18%)	1	1
	< -2—≥ -3 Z score	1(0.69)	144(99.31%)	0.27(0.03—2.4)	0.3(0.03–2.9)
	< -3 Z score	18(14.17)	109(85.83%)	5.7(1.9—16.9)	4.34(1.13–16.6)*
Weight/Height	≥ -2 Z score	10(4.35%)	220(95.65%)	1	1
	< -2- ≥ -3 Z scor	3(2.91%)	100(97.09%)	0.6(0.17—2.3)	0.87(0.18–4.26)
	< -3 Z score	10(12.35)	71(87.65%)	2.9(1.2—6.97)	1.02(0.3–3.5)
Isoniazid	Yes	4(2.48%)	157(97.52%)	0.35(0.2—1.02)	0.63(0.2–2.06)
Prophylaxis	No	19(7.51%)	234(92.49%)	1	1
Cotrimoxazole	Given	18(5.32%)	320(94.68%)	0.66(0.25—1.8)	0.27(0.08–0.87)*
Prophylaxis	Not given	5(6.67%)	70(93.33%)	1	1
Hemoglobin	≥ 10 g/dl	16(4.17%)	368(95.83%)	1	1
Level	< 10 g/dl	7(35%)	13(65%)	11(4.8—29)	3.7(1.1–12.7)*
CD4	≥ Threshold	11(3.35%)	317(96.65%)	1	1
Count	< Threshold	12(17.65%)	56(82.35%)	5.4(2.4—12)	4.86(1.9–12.7)*
WHO clinical	I&II	7(2.44%)	279(97.56%)	1	1
Stage	III&IV	16(12.5%)	112(87.5%)	5.2(2—12.6)	8.1(1.97–33)*
Regimen	Yes	9(3.33%)	261(96.67%)	0.25(0.11–0.59)	0.35(0.12–1.00)
Change	No	14(9.72%)	130(90.28%)	1	1

*Note*: *- predictors with *p*-value less than 0.05, *CHR* Crude hazard ratio, *AHR* Adjusted hazard ratio, *CI* Confidence interval

## Discussion

The study found that the overall mortality rate was 11.6 per 1000 child-year observation (95% CI: 7.7—17.5). The mortality rate observed in this study aligns with findings from Southwest Ethiopia (11.2 deaths per 1000 child-year observations) [18], Asia–Pacific (16.5 deaths per 1000 child years) [32], and the International Maternal Pediatric Adolescent AIDS Clinical Trials (IMPAACT) P1074 multicenter cohort studies in the United States (7 per 1000 child years) [33]. However, the result indicate a lower mortality rate compared to studies conducted in the Benishangul Gumuz region (54 deaths per 1000 child years) [34], Northwest Ethiopia (54 deaths per 1000 child years) [35], referral hospitals of Amhara regional state (44 deaths per 1000 child years) [25], selected public hospitals of the Addis Ababa city administration (4.98 deaths per 1000 child months) [36], the government ART centers in Mumbai, India (22.75 deaths per 1000 child years) [37], and Tanzania (135 per 1000 child years) [38].

Possible reasons for the variations among these studies could be the difference in health care awareness and knowledge level of the community, sample size, study period, and/or characteristics of the study participants, the difference in the adherence level of the ART, the difference in utilization of cotrimoxazole preventive therapy, the difference in a level of efforts to overcome adherence barriers regarding ART care and treatment, and the availability of advanced treatment modalities with low side effects.

In this study, children having weight for age < -3 Z score were almost 4.9 times more risk to death compared to children with weight for age > -2 Z score. This finding was in line with studies done in Arbaminch Town Southern Ethiopia [31], Wolaita zone southern Ethiopia [29], Debre Tabor and Dessie Northern Ethiopia [28], and Tanzania [39]. These could be due to undernutrition: there may be a delayed recovery from infections, an accelerated disease progression, a tendency for opportunistic infections, and a reduced ART response. Height for age was another predictor of mortality. This finding was in agreement with researches conducted in Benishangul Gumuz region [34], Amhara referral hospitals and India [25, 40]. These could be because undernutrition is a major predictor of treatment failure [41, 42], and undernourished children are at increased risk of dying as a result of the detrimental effects of undernutrition on ART treatment.

Children presented with hemoglobin level < 10 g/dl had a higher risk of death compared to children presented with hemoglobin level ≥ 10 g/dl. Previous study findings in Ethiopia [25, 29, 36, 43], Tanzania [39], and Kenya [44] had shown that low hemoglobin level was a significant risk of death among children on ART. These could be justified by the fact that when ART is started after a child experiences anemia from HIV infection, the child’s condition may only be partially cured, which could eventually lead to a low quality of life and mortality [45].

Baseline CD4 count was found significant predictor of mortality in this study. Children who were presented with a CD4 count below threshold at the time of ART initiation were approximately 4.7 times high risk to death compared to children with a baseline CD4 count greater than threshold. This finding was consistent with study findings in Ethiopia [25, 31], multiregional cohort analysis (Asia Pacific, East Africa, Southern Africa, West Africa) [46], India [40], and Tanzania [39]. A possible reason might be that low CD4/ immunodeficiency results in serious life-threatening opportunistic infections of the CNS, RS, and GI, as well as others [8] which in turn increases the risk of death in the early stages of treatment before responding to initiated ART.

An advanced WHO clinical stage of disease at the time of ART initiation was a predictor of mortality among undernourished children on ART. Children presented with advanced WHO disease stages (WHO stages III and IV) were at high risk of death compared to their counterparts who presented with mild WHO clinical stages I and II. Similar findings were reported in previous studies in Ethiopia [18, 25, 31], Vietnam [47], Swaziland [48], India [40], Cameroon [49], Tanzania [39], and South Africa [50]. These could be explained by the fact that in these children, antiretroviral therapy initiation could result in an immune reconstitution inflammatory syndrome due to severe immune cell depletion and progressive disease [8].

Children who took baseline cotrimoxazole preventive therapy were less likely to die for a shorter duration compared to their counterparts. The risk of death among children who took baseline cotrimoxazole preventive therapy was reduced by 73%. This finding was supported in previous studies in Ethiopia [31, 51, 52], Sub-Saharan Africa [53], and Asia [54]. The possible justification might be related to the therapy’s crucial role in preventing opportunistic infections and promoting immune recovery [55].

## Conclusion and recommendation

The incidence of mortality was determined in the study to be 11.6 per 1000 child years. Mortality was predicted by severe stunting, severe underweight, a low hemoglobin level, a low CD4 count, and WHO clinical stages III and IV. But the risk of death is reduced by starting cotrimoxazole preventative therapy early. The risk factors that result in a low survival status should be the primary focus of all concerned bodies, and early cotrimoxazole preventive treatment initiation is strongly recommended.

## Limitations of the study

Due to the secondary nature of the data, important nutritional factors like micronutrient deficiencies, feeding practices, food insecurity, wealth index, parental educational status, and combined nutritional status were not included in the study.

## Data Availability

Data set used in this study will be available from corresponding author on reasonable request.

## Abbreviations

AHR: Adjusted Hazard Ratio
AIDS: Acquired Immune Deficiency Syndrome
ART: Antiretroviral Therapy
CD4: Cluster of differentiation of cells
CI: Confidence interval
COVID: Corona Virus Disease
FMOH: Federal Ministry of Health
HA: Height for Age
HIV: Human Immunodeficiency Virus
HR: Hazard Ratio
IMPAACT: International Maternal Pediatric Adolescent AIDS Clinical Trials
IHN: Isoniazid
MRN: Medical Registration Number
OI: Opportunistic Infection
PLHIV: People Living with Human Immune Virus
PYO: Person Year Observation
RR: Relative Risk
SD: Standard Deviation
SPSS: Statistical Package for the Social Sciences
UNAIDS: Joint United Nations Program on HIV and AIDS
UNICEF: United Nations International Children’s Emergency Fund
WA: Weight for Age
WH: Weight for Height
